# Determinants of Fertility Intentions among South Koreans: Systematic Review and Meta-Analysis

**DOI:** 10.3390/bs14100939

**Published:** 2024-10-14

**Authors:** Eungyung Kim, Jee-Seon Yi

**Affiliations:** 1 Department of Nursing & Research Institute of Nursing Science, Chungbuk National University, Cheongju 28644, Republic of Korea; kyung11@chungbuk.ac.kr; 2 College of Nursing & Sustainable Health Research Institute, Gyeongsang National University, Jinju 52727, Republic of Korea

**Keywords:** fertility intention, meta-analysis, network analysis, South Korea, low fertility

## Abstract

(1) Background/objectives: This study aims to systematically review and conduct a meta-analysis of factors influencing fertility intentions among South Koreans. This research is crucial given South Korea’s lowest-in-the-world fertility rate of 0.72 in 2023, necessitating rapid and effective policies to address this demographic challenge; (2) Methods: Articles published from database inception through April 2024 were collected from five Korean databases using keywords based on the PEO (Population, Exposure, Outcome) framework. Following PRISMA guidelines, 35 articles were selected. The effect sizes and network of predictors related to fertility intention were analyzed using the R statistical package; (3) Results: A meta-analysis of the effect sizes of factors influencing fertility intentions revealed that the husband’s involvement in parenting (ESr = 0.131), women’s education level (ESr = 0.127), socioeconomic status (ESr = 0.116), and the expected gender of the child (ESr = 0.068) showed statistically significant positive effects. Conversely, women’s age (ESr = −0.175), parental stress (ESr = −0.146), and household labor ratio (ESr = −0.117) showed statistically significant negative effects. The network analysis further elucidated the complex interrelationships among these factors; (4) Conclusions: This study suggests the need for multifaceted policy approaches to address Korea’s low fertility, emphasizing promoting men’s participation in parenting, supporting women’s education and career development, reducing parenting stress, supporting work–family balance, and ensuring economic stability. These findings provide important insights for policymakers and researchers addressing the complex issue of low fertility in South Korea and may inform more effective interventions to boost fertility rates.

## 1. Introduction

Over the past 70 years, global fertility rates have more than halved, declining from approximately five children per woman in 1950 to 2.3 in 2021 [1]. South Korea stands at the forefront of this global demographic shift, exemplifying the challenges faced by many developed nations. The country’s total fertility rate (TFR) reached an unprecedented nadir of 0.72 in 2023, positioning it at the bottom of OECD rankings [2]. This figure not only undershoots the population replacement level of 2.1 [3] but also significantly trails behind other low-fertility countries such as Japan (1.3) and Italy (1.24) [4].

The gravity of Korea’s situation becomes even more apparent when viewed against the backdrop of global fertility trends. While many developed countries have experienced fertility declines, the rapidity and depth of Korea’s decrease are unparalleled. This precipitous drop poses formidable obstacles to demographic recovery and societal sustainability. The Korean Sustainable Development Goals, which are based on the United Nations Sustainable Development Goals framework, include addressing the issue of low fertility [5]. Despite various policy efforts under the Basic Plan for Low Fertility and Aged Society since 2006, these initiatives have not managed to significantly impact fertility rates [6,7]. The persistent downward trajectory, despite considerable governmental efforts, underscores the complexity of the issue and the need for innovative, multifaceted approaches to address this demographic challenge.

Fertility intentions are influenced by a complex interplay of factors. These include demographic aspects (e.g., delayed marriage, increased maternal age), socio-economic factors (e.g., child-rearing costs, work–family balance challenges), and cultural elements (e.g., gender equality, changing family values) [8,9,10]. The decline in fertility rates is particularly associated with factors like family gender equality, women’s status and empowerment, education, income, child-rearing practices, and women’s participation in economic activities [7,10,11,12].

Despite acknowledging the various factors influencing fertility intentions, much of the existing research on this topic has utilized a cross-sectional approach [7,11,13]. There is a lack of comprehensive analysis regarding how they interact and their cumulative impact on fertility intentions. Therefore, an integrated approach is essential to fully understand the interplay between these variables and their influence on fertility intentions. Furthermore, since the factors affecting fertility intentions may evolve over time, evaluating their effects using both current and long-term data is crucial. This approach will aid in developing policies that can effectively address changing social contexts.

This study employs both meta-analysis and network analysis. Meta-analysis systematically reviews existing studies to identify key influencing factors and their effects, providing valuable insights for more effective policy development [14]. Network analysis offers a deeper understanding of the complex interdependencies among factors and their indirect effects, serving as a tool for visualizing and analyzing relationships and interactions [15]. Since factors influencing fertility intentions are often interdependent, with one factor potentially impacting others, network analysis is particularly useful for gaining a deeper understanding of the intricate interactions affecting fertility intentions.

Thus, this study aims to systematically review research on the fertility intentions of South Korean women, identify relevant factors and effect sizes, and elucidate the relationships among these factors through network analysis.

## 2. Materials and Methods

### 2.1. Study Design

This study is a methodological systematic review and meta-analysis conducted to merge the effect sizes of factors influencing fertility intentions among Koreans according to the Population, Exposure, Outcome (PEO) framework. Additionally, this identified an Eigenvector centrality network of predictors that significantly influence fertility intention. This systematic review has been registered in the OSF Registries under the “Yi, J. (17 September 2024). Determinants of Fertility Intentions among South Korea: Systematic Review and Meta-Analysis. Retrieved from osf.io/epxy3” [16]. And, it was conducted rigorously with the PRISMA 2020 guidelines [17].

### 2.2. Eligibility Criteria and Outcome Variables

This study was conducted following the PRISMA (Preferred Reporting Items for Systematic Reviews and Meta-Analysis) guidelines [17], and the report was prepared by the PRISMA 2020 checklist [17]. This study applied the systematic review of association (etiology) method based on the Joanna Briggs Institute’s guideline [18]. The core question of the literature search was set to “What factors influence Koreans’ fertility intentions?” in accordance with the question format of PEO-SD (Population, Exposure, Outcome, and Study Design). The inclusion criteria were as follows: The study population (*p*) included Korean adults of reproductive age, typically between 15 and 49 years old; the exposure (E) included personal, social, economic, and cultural factors influencing fertility intention; and regarding outcome (O), the focus was on fertility intention. The study design (SD) involved cross-sectional survey studies, which included the published manuscript. Specific inclusion and exclusion criteria were as follows (Table 1). According to the inclusion and exclusion criteria presented in Table 1, five electronic domestic databases were searched, and papers published in English or Korean were selected.

### 2.3. Search Strategy

**Figure 1:**
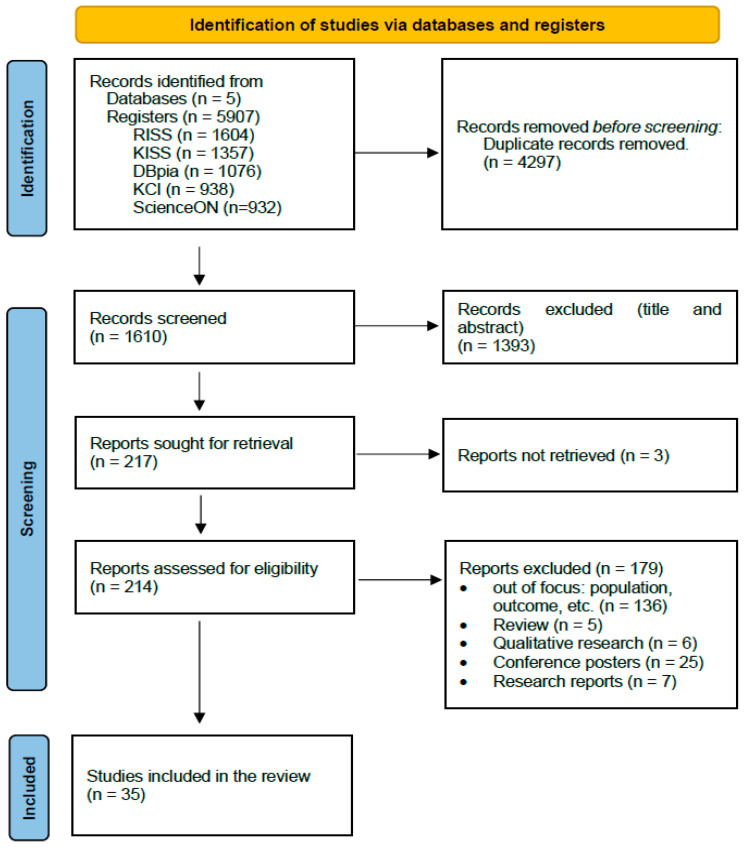
PRISMA flow diagram.

Preliminary searches were conducted following the above-listed criteria from 1 April 2024 to 22 June 2024 across e-journals and databases. The search strategy included five Korean domestic databases: the Research Information Sharing Service (RISS), Korean Studies Information Service System (KISS), DBpia (Database Periodical Information Academic), Korea Citation Index (KCI), and ScienceON. Searches for research articles written in Korean were conducted using the Korean search terms “childbearing possibility” OR “childbirth intention” OR “childbirth plan” OR “fertility plan” OR “fertility intention” in the title and abstract fields. For research articles written in English, searches were conducted using PubMed’s MeSH database with terms such as “fertility intention”, “childbearing intention”, or “childbirth intention” in the title and abstract fields. Two researchers independently extracted the data using tools such as the peer review of the electronic search strategy checklist to ensure the validity of the search. The PRISMA statement [17] proposes that the search process comprises three steps: identification, screening, and inclusion. All extracted data were consolidated using EndNote, and its automatic duplicate detection feature was employed to remove duplicates. After eliminating duplicate papers, the inclusion and exclusion criteria outlined in Table 1 were applied (Figure 1).

### 2.4. Data Extraction

After sharing the search formulas, the two researchers independently collected data and then collaboratively reviewed the papers to identify any discrepancies in the title, abstract, and full manuscript review process. We reached a consensus on inclusion and exclusion criteria through discussion. The independently collected papers were systematically summarized in Excel, following a step-wise method aligned with the review process. The researchers extracted paper data for analysis, categorizing them according to selection and exclusion reasons using numerical or color-coding systems. The initial evaluation involved screening the titles and abstracts of papers from the database search. Duplicate papers were further eliminated by sorting them by title and author using Excel 2019’s filtering function, ensuring a thorough removal of any remaining redundancies. Subsequently, a step-by-step review of the title, abstract, and full manuscript was conducted based on the inclusion and exclusion criteria outlined in Table 1. Papers that failed to meet these criteria were excluded, resulting in the final selection of papers for analysis. This study extracted the following data: author, publication year, sample size, study method, gender, marital status, employment status, living location, and number of children. Additionally, correlation coefficients or unstandardized coefficient (B) values and standard error values from regression analyses of variables affecting fertility intention were extracted, along with significant and non-significant results (*p*-values). For predictors related to fertility intention that presented only the correlation coefficients of a subdomain, the study replaced these with representative factor names and treated the original values as missing.

### 2.5. Quality Assessment

Each included article was independently reviewed twice for methodological quality by two research members using an adapted quality assessment tool for correlational studies used in previously published systematic reviews [19]. The tool was used to assess thirteen items. Twelve items were scored as either zero (=not met) or one (=met). One item, related to the measurement of fertility intention, was scored as two (=objective observation), one (=self-report), or zero (=not met). Studies were evaluated on sampling, statistical analysis, research design, and measurement, and scored as low (0–4), medium (5–9), or high quality (10–14).

### 2.6. Data Analysis

The characteristics of the studies were analyzed using SPSS statistical software (ver.29). In cases where the correlation coefficient (*r*) was not available, it was converted to r using a formula that utilizes sample size (*n*), unstandardized regression coefficient (*B*), and standard error (*SE*). The correlation coefficient (*r*) was derived from the unstandardized regression coefficient (*B*) and its standard error (*SE*) using the following transformation [20]:

$$r=\sqrt{\frac{{\left(B/SE\right)}^{2}}{{\left(B/SE\right)}^{2}+\left(n-p\right)}}\cdot sign\left(B\right)$$

where *n* is the sample size and *p* is the number of predictors in the model. When fertility intention was measured as a binary variable, the biserial correlation coefficient was used [21]. The effect sizes and network of predictors related to fertility intention were analyzed using the R statistical package (version 4.4.1). Since r is affected by the distribution of variance, it was converted to Fisher’s Z, which approximates a normal distribution, and the analysis results were then converted back to r for analysis (ESr) [22]. The final effect size interpretation followed Cohen’s criteria: ESr of 0.10 or less was interpreted as a small effect size, around 0.30 as a medium effect size, and 0.50 or more as a large effect size. The heterogeneity of the effect sizes for each factor was determined using Q and I^2^ statistics. A random-effects model was applied when I^2^ was 50% or higher and the *p*-value of Cochran’s Q was less than 0.05, indicating heterogeneity in effect sizes. In other cases, a fixed-effects model was applied. Statistical significance was determined using 95% confidence intervals (CIs), and an effect was considered significant if ‘0’ was not included in this interval [22]. To assess publication bias in results showing significant effect sizes through 10 or more studies, Egger’s test, funnel plot, and trim-and-fill method analyses were conducted. Egger’s test provides a statistical assessment of funnel plot asymmetry, which can indicate publication bias. The funnel plot offers a visual representation of this potential bias, while the trim-and-fill method estimates and adjusts for the number and outcomes of missing studies that might exist due to publication bias [23].

## 3. Results

### 3.1. Data Extraction Process

The total number of studies found in the database search was 5907 (1604 from RISS, 1357 from KISS, 1076 from DBpia, 938 from KCI, and 932 from ScienceON). Of these, the number of studies remaining after removing duplicate literature was 1610, with 4297 redundant papers in each database. After the exclusion of 3 studies for which the original text could not be found when reviewing all the databases together, 1607 studies remained. The final 35 studies were selected after removing 1572 studies by applying the selection and exclusion criteria again (Figure 1).

### 3.2. Characteristics of the Selected Studies and Participants

This study conducted a meta-analysis of 35 studies (with a total of 476,819 participants) that presented correlations between factors influencing fertility intentions. The publication years of the target studies ranged from 1 January 2010 to 31 March 2024. The study methods included cross-sectional surveys (15 studies) and secondary data analysis papers (20 studies). 98.6 percent of participants were female (470,138), and 98.7 percent were married (470,796). Twenty-two studies did not consider the employment status of the participants, and five studies sampled subjects nationwide. The measurement tool for fertility intention was a ‘binary scale’ in 19 studies and a ‘continuous scale’ in 16 studies. The most common type of study (22 studies) selected participants with one or more children (Table 2).

### 3.3. Methodological Quality

As a result of conducting a quality assessment on the 35 finally selected studies, the mean score for the 35 quantitative studies was 7.35. The selected studies were distributed between 6 and 10 points out of a total of 14 points. This corresponds to a medium to high-level score for all studies, indicating that all selected studies were evaluated as appropriate (see Supplementary Materials).

### 3.4. The Effect Sizes of Factors Influencing Fertility Intentions

**Figure 2:**
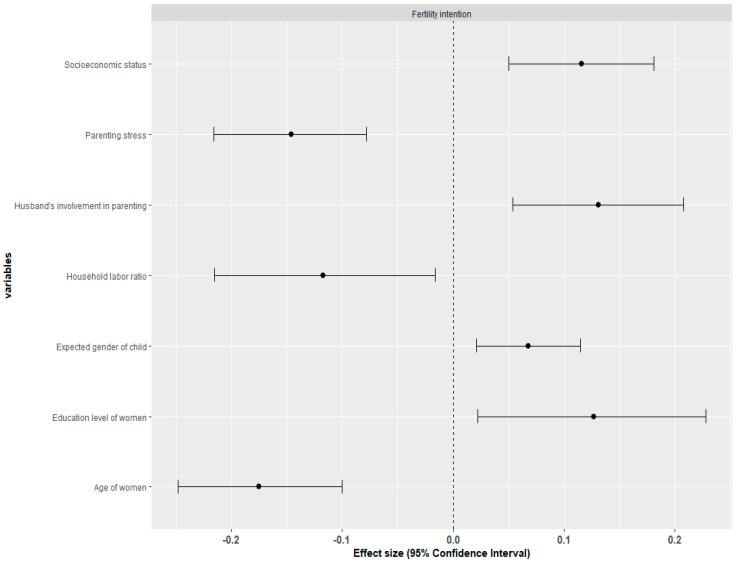
Effect sizes of related factors for fertility intention of Koreans.

To enhance the reliability of the study and the statistical significance of the results, the effect sizes of correlations were analyzed based on variables that included at least three studies, out of 35 papers that presented correlations with fertility intentions [24]. A meta-analysis of the effect sizes of factors influencing fertility intentions revealed that the husband’s involvement in parenting (ESr = 0.131), women’s education level (ESr = 0.127), socioeconomic status (SES) (effect size = 0.116), and the expected gender of the child (effect size = 0.068) showed statistically significant positive effects. Conversely, women’s age (effect size = −0.175), parental stress (effect size = −0.146), and household labor ratio (effect size = −0.117) showed statistically significant negative effects. On the other hand, factors such as marital satisfaction, maternal employment rate, marital conflict, emotional value of children, gender equality awareness, education level of men, self-efficacy, self-esteem, perception of parental responsibility, income, instrumental value of children, childcare expenses burden, depression of mother, and child-rearing expense income ratio (CEIR) did not significantly influence fertility intentions (Table 3, Figure 2).

### 3.5. Publication Bias

Regarding fertility intention, there were four factors with a positive influence and three factors with a negative influence that were significant variables. The application of Egger’s test, funnel plot, and trim-and-fill method to verify publication bias in the meta-analysis results for these variables recommends a minimum of 10 studies, so it was only conducted for the variables of husband’s involvement in parenting, age of women, and parenting stress. The Egger’s test for ‘Husband’s involvement in parenting’ (z = 0.28, *p* = 0.777) was not statistically significant (*p* > 0.05), the funnel plot was symmetrical, and the trim-and-fill analysis also indicated that there were 0 studies assumed to be missing, suggesting no publication bias. The Egger’s test for ‘age of women’ (z = −1.18, *p* = 0.239) was not statistically significant (*p* > 0.05), and the trim-and-fill analysis also indicated that there were 0 studies assumed to be missing, suggesting no publication bias. Although the funnel plot showed slight asymmetry, it was not statistically significant, so there was no evidence of publication bias. The Egger’s test for ‘parenting stress’ (z = −0.61, *p* = 0.542) was not statistically significant (*p* > 0.05), and the trim-and-fill analysis also indicated that there were 0 studies assumed to be missing, suggesting no publication bias. Although the funnel plot showed slight asymmetry, it was not statistically significant, so there was no evidence of publication bias.

For variables where publication bias could not be determined due to the small number of studies per variable, interpretation was done using the analyzed model. The expected gender of the child and SES were interpreted using the fixed model with I^2^ less than 50%, which assumes that these studies estimate relatively similar effect sizes [25]. This can be interpreted as the studies being conducted under very similar conditions with little variability between studies [26], thus showing narrow confidence intervals [27].

On the other hand, the husband’s involvement in parenting, women’s education level, age of women, parenting stress, and household labor ratio variables showed a random effect model. These significant variables reflect heterogeneity between studies [25], indicating that research was conducted under various conditions, showing wide confidence intervals, and the generalizability of research results can be considered higher than in the fixed effect model [27,28].

### 3.6. Network between Fertility Intention and Predictors

**Figure 3:**
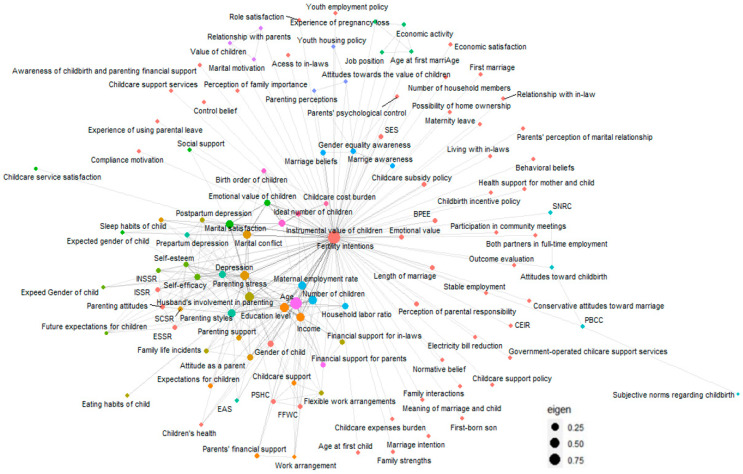
Network graph between fertility intention and predictors. CEIR = child-rearing expense income ratio; FFWC = family-friendly workplace culture; BPEE = burden of private education expenses; SES = socioeconomic status; SNRC = subjective norms regarding childbirth; PBCC = perceived behavioral control over childbirth; INSSR = instrumental social support from relatives; SCSR = social companionship support from relatives.

Figure 3 presents a network graph showing the eigenvector centrality of factors identified as predictors of fertility intention for Koreans. Eigenvector centrality demonstrates the importance of factors identified as predictors of fertility intention. It integrates both the frequency of relationships with variables significant to fertility intention and the connectivity with other related factors [29]. Therefore, the size of the node indicates the core factors influencing fertility intention. The interconnecting lines between variables represent statistically significant correlations. This network visualization provides a comprehensive overview of all variables demonstrating a significant impact on fertility intention, synthesized from 35 distinct studies. Among the 109 predictor variables of fertility intention, several factors stood out. These include age, husband’s involvement in parenting, number of children, education level, income, parenting styles, parenting support, gender of the child, maternal employment rate, marital satisfaction, marital conflict, parenting stress, self-efficacy, self-esteem, instrumental value of children, and child cost burden.

## 4. Discussion

This study systematically reviewed and conducted a meta-analysis of factors influencing fertility intentions among Koreans. The study’s findings identified both positive and negative factors that significantly influence fertility intentions, thereby providing critical implications for strategies aimed at increasing fertility rates. Since 2006, the South Korean government has been formulating and implementing quinquennial low fertility countermeasures in response to the declining birth rate. In the initial stages, these policies primarily emphasized increasing fertility rates through support mechanisms targeting infants and young children, with a significant focus on direct financial subsidies. However, recent policy shifts have prioritized respecting individual reproductive choices, enhancing overall quality of life, and promoting comprehensive structural reforms across various societal domains. The recent fourth quinquennial plan (2021–2025) has significantly expanded support measures across various domains. Notably, it has augmented financial assistance for childbirth and child-rearing expenses, expanded childcare services, improved parental leave policies, and promoted flexible work arrangements. Furthermore, the plan has increased housing supply and housing cost support for newlywed couples, extended coverage for infertility treatments, and enhanced tax benefits and priority housing allocation for multi-child households [30]. Despite these policy initiatives, the findings of this study reveal that fertility intentions are influenced by a complex array of factors, including the husband’s involvement in parenting, parenting stress, household labor ratio, women’s educational level, expected gender of the child, socioeconomic status, and maternal age. Notably, the results indicate that fertility intentions are more significantly impacted by sociocultural perceptions associated with fertility, such as the anticipated burden of domestic responsibilities and persisting son preference, rather than purely demographic or economic factors.

Firstly, the involvement of husbands in parenting was found to be the most significant positive factor affecting fertility intentions. This underscores the importance of gender equality within the household and shared parenting, suggesting that policies promoting paternity leave and the creation of family-friendly workplace cultures should be considered [31,32]. Countries with high fertility rates, such as Norway, Sweden, the Netherlands, and Denmark, have created environments that allow parents to spend more time with their children by expanding parental leave and reducing working hours. These countries have implemented institutional support measures such as early childhood care support, parental leave, and flexible working hours to realize practical gender equality [32,33]. This is supported by research showing that policies such as maternity and paternity leave, as well as parental leave, promote women’s participation in economic activities and men’s participation in child-rearing, thereby enhancing gender equality [34,35]. Such policy approaches also provide significant implications for establishing a gender-equal employment environment in Korea. Furthermore, countries like Finland and Iceland have achieved increased fertility rates and gender equality through comprehensive family support policies, which serve as reference models for Korea [36]. Therefore, it is essential to create gender-equal employment systems and a corporate culture supportive of child-rearing environments [36]. In particular, for dual-income couples, the husband’s participation in child-rearing has a positive impact on additional fertility intentions [37], highlighting the growing importance of policies that support work–family balance.

Secondly, the finding that women’s education level positively influences fertility intentions is consistent with previous studies [11]. This may be because highly educated women are likely to expect an equitable division of domestic labor at home [38], and they possess greater economic capability and confidence in raising children [11,39,40]. However, considering previous research [33] that suggests higher education levels among women may lead to delayed marriage and postponement or abandonment of childbirth due to participation in economic activities [41,42,43], policy approaches that support work–family balance and expand childcare infrastructure appear necessary.

Thirdly, SES and the expected gender of the child were also found to have positive effects on fertility intentions. This indicates that economic stability is a crucial factor in making decisions about childbirth [44,45], and it reflects the ongoing preference for male children in Korean society [46,47,48]. These phenomena can be explained by the cultural values and the rational choice theory. The cultural values can be explained that societal preferences regarding the gender of children influence fertility intentions [11,49,50], and the rational choice theory suggests that when economic resources are ample, fertility intentions increase [51]. When economic resources are sufficient, households can afford the costs associated with child-rearing, which contributes to a higher willingness to have more children [50].

On the other hand, the study found that women’s age was the most significant negative factor affecting fertility intentions. The association between age and fertility intentions is indisputable [11], particularly as higher age appears to have a negative impact on subsequent childbirth [52,53]. In 2022, the average age at first marriage in Korea was 33.7 years for men and 31.3 years for women [54], which is later compared to the OECD average of 30–32 years for men and 28–30 years for women [55]. This supports previous research suggesting that late marriage is one of the major causes of low birth rates [41]. The increasing age at marriage reflects broader social and economic trends, including prolonged educational pursuits and career development, which contribute to delaying family formation. Advanced maternal age is associated with increased risks in pregnancy and childbirth, which further complicates fertility intentions [53]. Given these factors, addressing the impact of delayed marriage on fertility rates requires a multifaceted approach, including policies that support work-life balance, provide financial incentives for families, and create an environment that accommodates both career and family aspirations. Such measures could potentially mitigate the negative effects of later marriage and advanced maternal age on fertility rates.

Parental stress is also one of the important factors influencing fertility intentions. Parents may experience difficulties, fatigue, and burnout during the child-rearing process, along with concerns about their child’s behavioral problems or anxiety about their parenting roles. These stress factors can reduce fertility intentions. Therefore, it is necessary to understand the various aspects of parental stress that can arise from multiple factors and to establish systematic counseling and psychological support programs to alleviate it. Expanding psychological support systems and stress management programs can reduce the burden on parents and contribute to increasing fertility intentions.

The household labor ratio was also found to have a negative impact on fertility intentions, indicating that the disproportionate burden of household chores and childcare on women hinders fertility intentions. The finding supports prior research [38,56,57,58,59], which posits that a more equitable distribution of domestic labor is associated with increased fertility intentions. In 2023, Korean women were responsible for 73.3% of household chores [60], which is higher than the 53.9% share of household labor handled by women compared to men in the United States [57]. Theoretical analysis indicates that working women experiencing significant work–family conflict often struggle to balance their professional and personal lives, leading to the perception that achieving both is unattainable. However, if husbands contribute to household chores, they can alleviate their wives’ work–family conflict, thereby effectively enhancing their willingness to have more children [61]. Therefore, it is necessary to expand social care services alongside the promotion of gender equality within households. To this end, initiatives such as campaigns and educational programs encouraging men’s participation in childcare, as well as incentive systems for companies to foster family-friendly cultures, should be considered.

Meanwhile, factors such as marital satisfaction, women’s employment status, marital conflict, emotional value of children, and gender equality awareness were not found to significantly influence fertility intentions. This finding differs from previous studies, which have shown that higher marital satisfaction or women’s employment increases fertility intentions [62], and that higher work–family conflict decreases fertility intentions [63,64]. Future research should analyze how these factors interact with other variables and explore their impact on specific groups or situations.

The network analysis results of this study show the complex interactions between factors influencing fertility intentions. Furthermore, these results elucidate additional factors influencing fertility intentions that were not prominent in the meta-analyses. These include experience with parental leave, support for infertile couples, adequacy of childcare facilities, satisfaction with care policies, gender equality awareness, and perceptions of marriage. These findings expand our understanding of the multifaceted determinants of fertility intentions beyond traditionally recognized factors. This suggests that fertility intentions are the result of complex interactions among personal, familial, and social factors [65,66].

Korean society has long been influenced by traditional ideologies that delineated distinct roles for men and women, with childcare primarily considered a female responsibility [67]. These findings suggest that to increase fertility rates, economic support policies should be complemented by educational initiatives aimed at shaping perceptions of marriage, childbearing, and gender equality, beginning prior to adolescence. Given the intrinsic connection between childbirth, childcare, and domestic responsibilities, multifaceted support policies at the national, local, and organizational levels are essential to alleviate women’s parenting stress and facilitate the fulfillment of their educational and professional aspirations. Moreover, these findings underscore the necessity of a long-term, strategic, and holistic approach at the national level to enhance fertility rates, encompassing initiatives to reshape perceptions among potential future parents and their families.

## 5. Conclusions

Study on the fertility intentions of Koreans has been consistently conducted, revealing that these intentions are influenced by complex interactions. The findings of this study suggest that increasing fertility intentions requires policy measures that extend beyond financial support, encompassing the promotion of gender-equitable employment environments, the fair distribution of household and childcare responsibilities, the reduction of parental stress, and the enhancement of gender equality awareness across society through comprehensive and integrated approaches.

This study believes it has represented the fertility intentions of Koreans of childbearing age by including a diverse range of participants, including unmarried men and women, couples with children, and childless couples. However, it failed to clearly delineate the factors influencing fertility intentions specific to each group’s characteristics. Future research should conduct in-depth analyses on the relationship between fertility intentions and actual fertility behaviors, differences in fertility intentions based on age and gender characteristics, and Korea’s particularities in comparison with other countries. Additionally, to assess the effectiveness of educational initiatives on perceptions of childbirth, marriage, and gender equality, longitudinal studies should be conducted to track changes in fertility intentions over time. Furthermore, qualitative research should be undertaken to explore deep-seated factors that are difficult to capture through quantitative analysis.

This study provides a comprehensive understanding of Koreans’ fertility intentions by analyzing 35 individual studies with a total of 476,819 participants. This approach offers statistical power and generalizability that is difficult to achieve in individual studies. Secondly, by including a wide range of variables that affect fertility intentions—such as personal factors (e.g., age, education level), family factors (e.g., husband’s participation in parenting, marital satisfaction), and socioeconomic factors (e.g., income, SES)—this study enables a comprehensive understanding of the complex mechanisms determining fertility intentions. Thirdly, this study does not merely analyze the effect sizes of individual variables but also visualizes the interactions and complex relationships between variables through network analysis. This helps in understanding the intricate interactions between factors that influence fertility intentions. Finally, by including studies on various population groups, such as university students, married women, and multicultural families, this study provides a comprehensive understanding of fertility intentions across different strata and groups in Korean society.

However, this study has several limitations. First, despite our best efforts to include all relevant studies that met the eligibility criteria, some studies may still have been missed due to limitations in the search keywords. Additionally, our search was limited to five South Korean databases, which may have led to an incomplete list of studies. Second, there may be differences in the measurement tools and operational definitions used in the included studies, so caution is needed in interpreting the results. Thirdly, the publication years of the studies included in the analysis span a wide range from 2010 to 2024. Therefore, changes in Korea’s socioeconomic conditions and fertility policies during this period may not have been fully reflected in the determinants of fertility intentions. Lastly, the exclusion of qualitative research is another limitation. As this meta-analysis only included quantitative studies, it did not incorporate insights from qualitative research that could explore the complex psychological and sociocultural factors influencing fertility intentions in greater depth. This may limit the overall understanding of the complex mechanisms underlying fertility intentions.

## Figures and Tables

**Table 1:** Study eligibility criteria.

PEO-SD	Inclusion Criteria	Exclusion Criteria
Participants	Adults between 15 and 49 years old living in Korea	Subjects outside the specified age range
Exposure	Individual characteristics, personal behaviors, and environmental factors influencing fertility intention Studies report correlation coefficient (r), unstandardized regression coefficient (B), standard deviation (SE), and sample sizes (n)	Missing r or B, SE, and n values
Outcomes	Fertility intention	Did not measure fertility intention as an outcome variable Studies in which fertility intention was measured but the effect size could not be calculated
Study design	Cross-sectional quantitative study	Studies that are not quantitative research Studies with low-quality (score 0–4)

**Table 2:** Characteristics of selected studies (K = 35, N = 476,819).

Category	Classification	Number of Studies K (%)	Number of Participants n
Publication year	2010–2014	13 (37.1)	12,088
	2015–2019	10 (28.6)	21,118
	2020–2024	12 (34.3)	443,613
Sample size	<500	18 (51.4)	4788
(person)	500–1999	9 (25.7)	7889
	≥2000	8 (22.9)	464,142
	M ± SD (range)	13,623.40 ± 55,404.89 (150–303,169)	
Instrument scale	Binary scale	19 (54.3)	463,000
	Continuous scale	16 (45.7)	13,819
Gender	Female	17 (48.6)	470,138
	Male	1 (2.9)	188
	Mixed	17 (48.6)	6493
Marital status	Marriage	21 (60.0)	470,796
	Singe	11 (31.4)	2860
	Mixed	3 (8.6)	3163
Employment	Yes	6 (17.1)	441,968
	No	7 (20.0)	1566
	Mixed	22 (62.9)	33,285
Location	Urban	13 (37.1)	141,183
	Urban and rural	17 (48.6)	11,961
	Nationwide	5 (14.3)	324,834
Number of children	0	11 (31.4)	3147
	≥1	22 (62.9)	470,997
	Mixed	2 (5.7)	2675
Total		35 (100.0)	476,819

A paper has been treated two studies because the data by distinguishing between the waves of data collection.

**Table 3:** Effect sizes of factors for fertility intentions and intent to have more children.

Classification	Variables	K (n)	ESr	95% CI Lower, Upper	Z (*p*)	Heterogeneity				Analyzed Model
						Tau^2^	I^2^ (%)	Q	df (*p*)	
Significant	Husband’s involvement in parenting *	10 (8862)	0.131	0.054, 0.208	3.31 (<0.001)	0.022	87.3	70.57	9 (<0.001)	Random
positive	Education level of women *	8 (150,739)	0.127	0.022, 0.228	2.38 (0.017)	0.021	94.5	128.43	7 (<0.001)	Random
effect	Expected gender of child *	8 (7889)	0.068	0.021, 115	2.83 (0.005)	0.003	18.42	18.42	7 (0.010)	Fixed
	SES *	3 (1452)	0.116	0.050, 0.181	3.45 (<0.001)	0.001	29.8	2.85	2 (0.241)	Fixed
Significant	Age of women *	15 (163,720)	−0.175	−0.248, −0.100	−4.52 (<0.001)	0.022	99.5	2908.99	14 (<0.001)	Random
negative	Parenting stress *	10 (9656)	−0.146	−0.216, −0.078	−4.17 (<0.001)	0.012	91.9	98.42	9 (<0.001)	Random
effect	Household labor ratio *	3 (8962)	−0.117	−0.215, −0.016	−2.27 (0.023)	0.005	67.2	6.10	2 (0.047)	Random
Non-significant	Marital satisfaction	9 (15,482)	0.061	−0.001, 0.121	1.96 (0.050)	0.008	92.5	106.00	8 (<0.001)	Random
positive	Maternal employment rate	7 (11,606)	0.009	−0.010, 0.269	0.88 (0.377)	0.065	98.6	440.71	6 (<0.001)	Random
factors	Marital conflict	6 (5831)	0.018	−0.091, 0.126	0.33 (0.745)	0.017	93.1	72.02	5 (<0.001)	Random
	Emotional value of children	6 (5987)	0.062	−0.060, 0.131	1.66 (0.096)	0.007	78.9	23.68	5 (<0.001)	Random
	Ideal number of children	4 (3804)	0.085	−0.012, 0.181	1.71 (0.087)	0.009	87.6	24.10	3 (<0.001)	Random
	Gender equality awareness	4 (829)	0.211	−0.098, 0.483	1.35 (0.178)	0.097	94.2	51.34	3 (<0.001)	Random
	Education level of men	3 (11,251)	0.051	−0.068, 0.169	0.84 (0.401)	0.010	94.2	34.27	2 (<0.001)	Random
	Self-efficacy	3 (4452)	0.018	−0.061, 0.097	0.45 (0.651)	0.004	84.8	13.20	2 (0.001)	Random
	Self-esteem	3 (4452)	0.008	−0.077, 0.092	0.18 (0.854)	0.004	81.4	10.77	2 (0.005)	Random
	Perception of parental responsibility	3 (3177)	0.028	−0.025, 0.080	1.03 (0.305)	0.001	47.6	3.82	2 (0.148)	Fixed
Non-significant	Income	10 (155,653)	−0.008	−0.063, 0.047	−0.28 (0.782)	0.007	95.0	178.47	9 (<0.001)	Random
negative	Instrumental value of children	7 (6283)	−0.001	−0.115, 114	−0.01 (0.995)	0.023	90.7	64.46	6 (<0.001)	Random
factors	Childcare expenses burden	5 (3480)	−0.029	−0.123, 0.066	−0.59 (0.552)	0.009	82.7	23.11	4 (<0.001)	Random
	Depression of mother	4 (6530)	−0.028	−0.119, 0.108	−0.10 (0.920)	0.012	95.3	63.83	3 (<0.001)	Random
	CEIR	3 (3177)	−0.059	−0.129, 0.011	−1.64 (0.100)	0.003	78.2	9.18	2 (0.010)	Random

* = significant factor; CEIR = child-rearing expenses to income ratio; CI = confidence interval; df = degree of freedom; ESr = correlational effect size; I^2^ = I^2^ test of quantifying heterogeneity; K = number of studies; Q = Q test heterogeneity; SES = socioeconomic status; Z = standard score.

## Data Availability

The data of the findings in this study can be available from the research team upon reasonable request.

## Supplementary Materials

The following supporting information can be downloaded at: https://www.mdpi.com/article/10.3390/bs14100939/s1, Target paper for analyses [31,32,37,40,41,44,46,56,57,68,69,70,71,72,73,74,75,76,77,78,79,80,81,82,83,84,85,86,87,88,89,90,91,92].
