# Supplementary material for: Determinants of Fertility Intentions among South Koreans: Systematic Review and Meta-Analysis
Source: Behav Sci (Basel). 2024 Oct 14;14(10):939. doi: 10.3390/bs14100939 (PMC11505273; doi:10.3390/bs14100939)
Supplement: Supplementary file 1 [file behavsci-14-00939-s001.zip › behavsci-3234368-supplementary.pdf]

Section and Topic	Item #	Checklist item	Location where item is reported
**TITLE**			
Title	1	Identify the report as a systematic review.	P.1
**ABSTRACT**			
Abstract	2	See the PRISMA 2020 for Abstracts checklist.	p.1
**INTRODUCTION**			
Rationale	3	Describe the rationale for the review in the context of existing knowledge.	P.2
Objectives	4	Provide an explicit statement of the objective(s) or question(s) the review addresses.	p.2
**METHODS**			
Eligibility criteria	5	Specify the inclusion and exclusion criteria for the review and how studies were grouped for the syntheses.	p.2
Information sources	6	Specify all databases, registers, websites, organisations, reference lists and other sources searched or consulted to identify studies. Specify the date when each source was last searched or consulted.	p.2,3,4
Search strategy	7	Present the full search strategies for all databases, registers and websites, including any filters and limits used.	p.3
Selection process	8	Specify the methods used to decide whether a study met the inclusion criteria of the review, including how many reviewers screened each record and each report retrieved, whether they worked independently, and if applicable, details of automation tools used in the process.	p.3
Data collection process	9	Specify the methods used to collect data from reports, including how many reviewers collected data from each report, whether they worked independently, any processes for obtaining or confirming data from study investigators, and if applicable, details of automation tools used in the process.	p.3
Data items	10a	List and define all outcomes for which data were sought. Specify whether all results that were compatible with each outcome domain in each study were sought (e.g. for all measures, time points, analyses), and if not, the methods used to decide which results to collect.	p.4
	10b	List and define all other variables for which data were sought (e.g. participant and intervention characteristics, funding sources). Describe any assumptions made about any missing or unclear information.	N/A
Study risk of bias assessment	11	Specify the methods used to assess risk of bias in the included studies, including details of the tool(s) used, how many reviewers assessed each study and whether they worked independently, and if applicable, details of automation tools used in the process.	p.5
Effect measures	12	Specify for each outcome the effect measure(s) (e.g. risk ratio, mean difference) used in the synthesis or presentation of results.	p.5
Synthesis methods	13a	Describe the processes used to decide which studies were eligible for each synthesis (e.g. tabulating the study intervention characteristics and comparing against the planned groups for each synthesis (item #5)).	P.2,3
	13b	Describe any methods required to prepare the data for presentation or synthesis, such as handling of missing summary statistics, or data conversions.	N/A
	13c	Describe any methods used to tabulate or visually display results of individual studies and syntheses.	p.5
	13d	Describe any methods used to synthesize results and provide a rationale for the choice(s). If meta-analysis was performed, describe the model(s), method(s) to identify the presence and extent of statistical heterogeneity, and software package(s) used.	p.5
	13e	Describe any methods used to explore possible causes of heterogeneity among study results (e.g. subgroup analysis, meta-regression).	p.5
	13f	Describe any sensitivity analyses conducted to assess robustness of the synthesized results.	p.5
Reporting bias assessment	14	Describe any methods used to assess risk of bias due to missing results in a synthesis (arising from reporting biases).	p.5
Certainty assessment	15	Describe any methods used to assess certainty (or confidence) in the body of evidence for an outcome.	p.5
**RESULTS**			
Study selection	16a	Describe the results of the search and selection process, from the number of records identified in the search to the number of studies included in the review, ideally using a flow diagram.	p.4

Section and Topic	Item #	Checklist item	Location where item is reported
	16b	Cite studies that might appear to meet the inclusion criteria, but which were excluded, and explain why they were excluded.	N/A
Study characteristics	17	Cite each included study and present its characteristics.	p.6/supp
Risk of bias in studies	18	Present assessments of risk of bias for each included study.	p.6/supp, 10
Results of individual studies	19	For all outcomes, present, for each study: (a) summary statistics for each group (where appropriate) and (b) an effect estimate and its precision (e.g. confidence/credible interval), ideally using structured tables or plots.	p.8,9
Results of syntheses	20a	For each synthesis, briefly summarise the characteristics and risk of bias among contributing studies.	p.6/supp
	20b	Present results of all statistical syntheses conducted. If meta-analysis was done, present for each the summary estimate and its precision (e.g. confidence/credible interval) and measures of statistical heterogeneity. If comparing groups, describe the direction of the effect.	p.6/supp,7,8,9
	20c	Present results of all investigations of possible causes of heterogeneity among study results.	p.10
	20d	Present results of all sensitivity analyses conducted to assess the robustness of the synthesized results.	N/A
Reporting biases	21	Present assessments of risk of bias due to missing results (arising from reporting biases) for each synthesis assessed.	N/A
Certainty of evidence	22	Present assessments of certainty (or confidence) in the body of evidence for each outcome assessed.	p.6,7,8,9,10
**DISCUSSION**			
Discussion	23a	Provide a general interpretation of the results in the context of other evidence.	p.12,13,14,15
	23b	Discuss any limitations of the evidence included in the review.	p.14,15
	23c	Discuss any limitations of the review processes used.	p.14,15
	23d	Discuss implications of the results for practice, policy, and future research.	p.13,14,15
**OTHER INFORMATION**			
Registration and protocol	24a	Provide registration information for the review, including register name and registration number, or state that the review was not registered.	p.2
	24b	Indicate where the review protocol can be accessed, or state that a protocol was not prepared.	p.2
	24c	Describe and explain any amendments to information provided at registration or in the protocol.	N/A
Support	25	Describe sources of financial or non-financial support for the review, and the role of the funders or sponsors in the review.	p.15
Competing interests	26	Declare any competing interests of review authors.	p.15
Availability of data, code and other materials	27	Report which of the following are publicly available and where they can be found: template data collection forms; data extracted from included studies; data used for all analyses; analytic code; any other materials used in the review.	p.15

From: Page MJ, McKenzie JE, Bossuyt PM, Boutron I, Hoffmann TC, Mulrow CD, et al. The PRISMA 2020 statement: an updated guideline for reporting systematic reviews. BMJ 2021;372:n71. doi: 10.1136/bmj.n71

Supplementary

[Target paper for analysis]

1. Kim, E-J., Chung, H., & Jeong, Y-J. (2022). Effects of the value on children, the motivation for marriage, the relationship with parents on the will to have a child: Multi-group path analysis by gender. Journal of Families and Better Life, 40(1), 27-39.

2. Lee, E-J., Hong, S-J., & Kim, S-H. (2022). Factors affecting childbirth will of university students. Journal of the Korea Academia-Industrial cooperation Society, 23(11), 684-692. https://doi.org/10.5762/KAIS.2022.23.11.684

3. Hong, S-H. (2020). Factors related to the willingness to have a child, parental age at first child's birth, and the planned number of children among men and women. Journal of Family Resource Management, 24(2), 69-87. https://doi.org/10.22626/jkfrma.2020.24.2.004

4. Lee, I-S. (2022). The influence of the intention of childbirth and family strengths on marriage value of nursing students. Journal of the Korea Academia-Industrial cooperation Society, 23(3), 78-86. https://doi.org/10.5762/KAIS.2022.23.3.78

5. Choi, J. H., & Ahn, S. H. (2018). The impact of family-friendly policies and paternal participation in child-care on married women's willingness to have an additional child. Journal of Families and Better Life, 36(1), 75-85.

6. Park, J. (2020). Structural relationship analysis of gender equality consciousness, perceptions of parenthood and procreation consciousness in 2030 generations. The Journal of Humanities and Social Science, 11(6), 2349-2364. https://doi.org/10.22143/HSS21.11.6.166

7. Lee, J., Cho, H., & Choi, H. (2012.). The differences in offspring birth plan of working mothers with one child and its effects on parenthood. International Journal of Child Care and Education, 6(1), 125-141.

8. Jung, Y., Kim, H., & Jung, S. (2013). A study on factors affecting family planning decisions of mothers who have 2 years old children: Focusing on parents' values on children and characteristics of couples. The Korean Journal of the Human Development, 20(2), 185-203.

9. Hyun, J-H. (2019). The effect of the parental non-maternal perspective of child rearing on subsequent birth will. Korea Journal of Child Care and Education, 118(9), 101-122.

10. Lee, S., & Lee, S. (2023). Effects of procreation consciousness and parenting skills belief on perception of parenthood among college students. The Journal of Humanities and Social Science, 14(2), 267-280. https://doi.org/10.22143/HSS21.14.2.19

11. Kim, S-J., & Cho, Y. (2022). Factors affecting the additional fertility intentions among dual-earner couples in Seoul, South Korea: Focusing on the experiences and expectations regarding prenatal policies. Korean Journal of Family Social Work, 69(3), 97-122. https://doi.org/10.16975/kjfsw.69.3.4

12. Kang, Y-S., & Kwak, S-J. (2018). A study on the influence on a follow-up childbirth plan by a mother's family interaction and her sufficiency awareness of a child care and education facilities: With focus on mediated effect of value of children. Early Childhood Education Research & Review, 22(3), 299-320.

13. Kim, K. S., & Kim, J. W. (2019). The impact of college students' gender equality consciousness to childbirth will: Focused on mediating about marriage awareness. Journal of Learner-Centered Curriculum and Instruction, 19(13), 69-88. https://doi.org/10.22251/jlcci.2019.19.13.69

14. Kim, J-Y., & Kim, Y-J. (2022). Factors influencing fertility intentions of unmarried men and women – Application of theory of planned behavior. Journal of Industrial Convergence, 20(5), 93-100. https://doi.org/10.22678/JIC.2022.20.5.093

15. Lee, M. J., Kim, K. H., & Lee, J. Y. (2012). A longitudinal study on moderating variables for following childbirth intention. Korean Journal of Child Education and Care, 12(2), 265-286.

16. Park, H. J., & Moon, H. J. (2017). The effects of child care service satisfaction and social support on mothers' intention of second childbirth: The mediating effect of marital satisfaction. The Journal of Korea Open Association for Early Childhood Education, 22(3), 25-43. https://doi.org/10.20437/KOAECE22-3-02

17. Seo, M. (2011). The effects of parents' psychosocial characteristics on follow-up planned childbirth: Focused on family with children. International Journal of Child Care and Education, 5(1), 127-148.

18. Kang, H-G., Shim, D., Pack, Y., & Yi, S. (2014). The impact of regional economic characteristics and life cycle on mothers' intention of having additional children: A study combining microscopic and macroscopic variables. International Journal of Child Care and Education, 8(2), 175-208.

19. Kim, K. S., & Lee, H. J. (2019). The effects of marriage awareness, career awareness, and gender equality to childbirth will among college students. Journal of Learner-Centered Curriculum and Instruction, 19(11), 77-98. https://doi.org/10.22251/jlcci.2019.19.11.77

20. Lee, S., Lee, S., & Moon, S. (2017). The study on the effect of double responsibility of elderly and child care on the future childbirth intention: Focusing on moderating effect of the presence of elderly care burden on the relationship between the number of children within household and the future fertility intention. Family and Culture, 29(3), 111-138.

21. Jang, H-J., & Lee, Y. J. (2023). The effect of university students' personal values and fertility promotion policy perception on childbearing willingness. The Journal of the Convergence on Culture Technology, 9(2), 83-90. https://doi.org/10.17703/JCCT.2023.9.2.83

22. Lim, H-J., Lee, D-K., & Choi, H-J. (2011). An examination of the influence of the variables of social support of friends and relatives, parental characteristics, and mother's emotional characteristics on planning for second childbirth. Journal of Early Childhood Education, 31(6), 167-189.

23. Lee, H. M. (2012). A study on the factors affecting additional birth plan: Focused on Korean households with infants. Korea Journal of Child Care and Education, 3(1), 1-31.

24. Kong, T-H., & Lim, J-D. (2011). Recognition on the birth bringing-up of fertile-women. The Korean Journal of Health Service Management, 5(3), 41-52.

25. Lee, J. W., Kim, K. E., & Lim, W. (2015). Family and child factors affecting the second childbirth plan in one child family: Focus on the family environments and children's daily habits. International Journal of Child Care and Education, 9(2), 1-22.

26. Lim, S. Y. (2016). Factors influencing the intention for additional childbirth among married couples. International Theological Journal, 15, 486-511.

27. Jeon, N. R., & Cho, B. H. (2012). A study on why married men decide to have additional children. Journal of Korean Child Care and Education, 8(1), 125-143.

28. Ding, J., Chin, M., & Ok, S. (2018). Factors affecting the intention of Chinese and Vietnamese migrant women to have a second child: Comparison between the "National Survey on the Multi-Cultural Families" of 2009 and 2015. Journal of Family Relations, 23(2), 133-155. https://doi.org/10.21321/jfr.23.2.133

29. Hong, S., Son, S., & Choi, J. (2023). Factors associated with the possibility of marriage and childbearing among never married young adults in Korea. Human Ecology Research, 61(2), 183-194. https://doi.org/10.6115/her.2023.013

30. Lim, H-J., Lee, D-K., & Choi, H-J. (2011). Using a structural equation approach to determine how a couple's characteristics, childbirth-related motions and a mother's emotional characteristics affect planning for second

childbirth. Journal of Early Childhood Education, 31(5), 183-202.

31. Lee, H., & Choi, Y. (2015). The effects of informal care resources on women's fertility intention. *Family and Culture, 27*(2), 262-288. https://doi.org/10.21478/family.27.2.201506.009
32. Lee, H., & Choi, Y. (2015). The effects of informal care resources on women's fertility intention. *Family and Culture, 27*(2), 262-288. https://doi.org/10.21478/family.27.2.201506.009
33. Ha, M. (2020). The effects of parenting stress and paternal participation on the number of child in follow-up childbirth plan of mothers: For mothers with first-born child. *Journal of Learner-Centered Curriculum and Instruction, 20*(3), 1139-1155. https://doi.org/10.22251/jlcci.2020.20.3.1139
34. Han, K. M., & Jung, H. S. (2010). The factors affecting the fertility intention in general hospital nurses based on the theory of planned behavior. *Korean Journal of Occupational Health Nursing, 19*(1), 41-49.
35. Park, S. Y., & Hwang, M. J. (2022). Factors on childbirth intention of working married women in Korea. *Journal of Public Society, 12*(4), 48-70.

Table 1. Quality Assessment and Validity Tool for Correlational Studies

Study ID	Design		Sample				Measurement					Statistical analysis		Total score
	1	2	3	4	5	6	7	8	9	10	11	12	13	
1	1	0	0	1	1	1	1	1	1	1	0	1	0	9
2	1	0	1	0	1	1	0	0	1	1	0	1	0	7
3	0	1	1	1	1	1	0	0	1	0	0	0	0	6
4	1	0	1	0	1	1	1	1	1	1	0	1	0	9
5	1	0	0	1	1	1	0	0	1	1	0	1	0	7
6	1	0	0	1	1	1	1	1	1	1	0	1	0	9
7	0	1	1	1	1	1	0	0	1	1	0	0	0	7
8	0	1	1	1	1	1	0	0	1	0	0	1	0	7
9	1	0	1	0	1	1	1	1	1	1	0	1	0	9
10	1	0	1	1	1	1	0	1	1	1	0	1	0	9
11	0	1	1	1	1	1	0	0	1	0	0	0	0	6
12	0	1	1	1	1	1	1	1	1	0	0	1	0	9
13	1	0	0	1	1	1	1	1	1	1	0	1	0	9
14	0	0	0	0	1	1	1	1	1	1	1	1	0	8
15	0	1	1	1	1	1	0	0	1	1	0	1	0	8
16	1	0	0	1	1	1	0	0	1	1	0	1	0	7
17	0	1	1	1	1	1	0	0	1	1	0	1	0	8
18	0	1	1	1	1	1	1	1	1	1	0	0	0	9
19	0	0	0	1	1	1	0	0	1	1	0	1	0	6
20	0	1	1	1	1	1	1	1	1	0	0	1	0	9
21	1	0	1	0	1	1	1	1	1	1	0	1	0	9
22	0	1	1	1	1	1	0	0	1	1	1	1	0	9
23	0	1	1	1	1	1	0	0	1	0	0	1	0	7
24	1	0	0	0	1	1	0	1	1	1	0	0	0	6
25	1	0	1	1	1	1	1	0	1	1	0	0	0	8
26	1	0	0	1	1	1	0	0	1	1	0	0	0	6
27	0	1	1	1	1	1	0	0	1	1	0	0	0	7
28	0	1	1	1	1	1	0	0	1	0	0	0	0	6
29	0	1	1	1	1	1	0	0	1	1	1	1	0	9
30	0	1	1	1	1	1	0	0	1	0	0	0	0	6
31	0	1	1	1	1	1	0	0	1	0	0	0	0	6
32	0	1	1	1	1	1	0	0	1	1	0	1	2	10
33	1	0	0	0	1	1	1	1	1	1	1	1	0	9
34	0	1	1	1	1	1	0	0	1	0	0	1	0	7
35	0	1	1	1	1	1	0	0	1	1	1	1	0	9
Total	14	19	25	28	35	35	12	13	35	25	5	24	2	7.35

*Study ID=List number of target paper for analysis
